# Sterilized Processed Cheese: Principles, Technological Aspects, and Properties: A Review

**DOI:** 10.3390/foods14061072

**Published:** 2025-03-20

**Authors:** Zuzana Lazárková, Eva Lorencová, Markéta Pětová, Martin Novotný, Richardos Nikolaos Salek

**Affiliations:** 1 Department of Food Technology, Faculty of Technology, Tomas Bata University in Zlin, nam. T.G. Masaryka 5555, 760 01 Zlin, Czech Republic; lorencova@utb.cz (E.L.); rsalek@utb.cz (R.N.S.); 2 Department of Logistics, Faculty of Military Leadership, University of Defence, Kounicova 65, 662 10 Brno, Czech Republic; marketa.petova@unob.cz (M.P.); martin.novotny5@unob.cz (M.N.)

**Keywords:** sterilized processed cheese, thermosterilization, long-term storage, processed cheese quality, Maillard reaction complex, lipid oxidation reactions

## Abstract

Sterilized processed cheese is a dairy product with prolonged shelf life compared to regular processed cheese. The extension of durability is made possible by the thermosterilization of processed cheese, while regular processed cheese is submitted to pasteurization process during manufacturing. Sterilized processed cheese can be classified as long-life foods and their shelf life may reach up to 24 months, if stored at ambient temperature (approx. 25 ± 1 °C). This fact is an advantage over regular processed cheese, which has a shelf life of only around 6 months. Sterilized processed cheese finds application in everyday life when refrigeration facilities are not available; i.e., it is intended for regular retail. However, their most important use is for storage in state material reserves and, moreover, for catering for members of the armed forces and/or members of the integrated rescue system. This review aimed to gather general information on sterilized processed cheeses, their characterization, usage and production. Furthermore, the review discusses the principles of sterilization and factors affecting the course of sterilization focusing on the setting the sterilization limits and sterilization parameters in order to maximize end-product quality. Moreover, last part of the review is devoted to the effect of sterilization and long-term storage on the qualitative parameters of sterilized processed cheese.

## 1. Introduction

Processed cheese (PC) can be characterized as a complex, multi-component system of dairy origin that can be described as a stable oil-in-water emulsion. PC is a dairy product made by blending natural cheese of different degrees of maturity in the presence of emulsifying salts and other dairy and nondairy ingredients. Dairy raw materials include the natural cheeses (Dutch-type, Swiss-type, Cheddar, Mozzarella, white brined, quark), rework (PC already melted), sweet or acid casein, caseinates, milk powder, buttermilk powder, whey powder or whey protein powder (or whey protein concentrates and isolates), butter or cream. Non-dairy raw materials include, for example, stabilizers, preservatives, hydrocolloids, emulsifiers, colorants, acidulants, salt, spices and flavorings, emulsifying salts and, of course, water. Stabilizers are added for technological reasons (ability to bind water and influence/stabilize consistency), especially to low-fat products. Furthermore, the inclusion of these additives in the raw material composition is particularly necessary when substituting traditional raw materials. Stabilizers, often hydrocolloids, are usually polysaccharide or protein-based additives (e.g., carrageenan, alginate, locust bean gum, guar gum, arabic gum, xanthan gum, pectin, gelatin). Blending of all raw materials is followed by heating and continuous mixing to create a smooth and homogeneous cheese matrix [1,2,3]. Adding emulsifying salts (mainly sodium phosphates, diphosphates, polyphosphates, and citrates) enhances the emulsifying capacity of proteins (i.e., caseins), improving their ability to act as emulsifiers. These salts sequester calcium resulting in a decrease in the concentration of free calcium ions and dissociation of the casein micelles into small clusters, thereby causing enhanced hydration and voluminosity of the caseins [4,5]. In Regulation (EC) No 1333/2008 of the European Parliament and of the Council on food additives, as amended, emulsifying salts are defined as substances which convert the proteins contained in cheese into a dispersible form in order to homogeneously distribute the fats and other ingredients [6].

Compared to natural chesses, PC has prolonged shelf life. In particular, PC durability can reach up to approx. 6 months at refrigeration temperatures [7]. To lengthen the shelf life up to 24 months at ambient temperature, it is possible to use thermosterilization (hereinafter referred to as sterilization) in hermetically closed containers. The application of heat is both an important method of preserving foods and a means of developing texture, flavor and color. Generally, it has long been recognized that thermal technologies must ensure the safety of food without compromising food quality. Sterilized processed cheese (SPC) is intended as food provisions for army members (both in peacetime and crisis/emergencies), for storage in state material reserves and also for regular retail [8,9,10].

Sterilization heating can significantly affect all the major components of PC; i.e., proteins, lipids and carbohydrates. Among the most important reactions are the Maillard reaction complex (MRC) and the lipid oxidation reaction (LOR) leading to changes in color, consistency, flavor and thermolabile biologically active substances. However, the organoleptic, nutritional and functional properties of foods treated with high temperatures should be maintained as much as possible [9,11]. Although sterilization can guarantee health safety of the product, even sterilized food is not completely stable and its long-term storage would be connected with physico-chemical changes, especially when elevated temperatures are used. Similar changes to those mentioned for the effect of sterilization can be expected during storage of SPC [12,13].

Currently, a detailed review paper on the sterilized processed cheese is missing from the available scientific literature. Thus, this work is aimed to summarize and describe the existing knowledge of the characterization, production and usage of SPC and, furthermore, on the effect of sterilization and long-term storage on SPC quality. The year range of reports covered in this review is 1992–2024, but most of the studies are from the last 15 years. The literature search strategy used was based on the Web of Science and SCOPUS database websites by using various queries (e.g., processed cheese, sterilized processed cheese, sterilization, thermosterilization, storage, long-term storage, durable dairy foods, sterilized dairy foods, UHT-milk, in-container sterilization, UHT sterilization, Maillard reaction, lipid oxidation, TBARS, etc.

## 2. Sterilized Processed Cheese

### 2.1. Usage of Sterilized Processed Cheese

In recent decades, the world has been threatened by both natural (natural disasters such as floods, earthquakes, avalanches, wildfires, tornadoes, tsunamis etc., mass infections) and anthropogenic (operational accidents, state of war) emergencies with the possibility of rapid escalation. Dealing with such situations requires advanced crisis management approaches. A crucial task for organizations that deploy rescue teams is to ensure the ability to provide logistical support to humanitarian and/or military missions [14]. In the first days of deployment of soldiers, integrated rescue system members or humanitarian workers, the so-called packaged food rations (previously referred to as combat or canned food rations) may be used as part of the logistical support of the respective operation. This is a full day’s ration consisting of food and ready meals which can be consumed partially cooked (heated) or cold and is issued in cases where it is not possible to provide a soldier with a hot meal. The primary objective of introducing packaged food rations into armies is to provide adequate food in a single ration for 24 h to ensure physical performance and cognitive function, whether during routine exercise or operational deployment [8,15].

The requirements for packaged batches of food are framed in the NATO standardization agreement STANAG 2937 [16] and specified in the follow-up standard AMedP-1.11 [17], which requires a minimum shelf life of all ingredients of at least 24 months at 25 °C. However, missions do not only take place in countries with temperate climates; subtropical and tropical climate zones must also be considered. Given the long distances from home bases, transport can take weeks. Food is thus routinely exposed to temperatures higher than the required 25 °C during transport and subsequent storage. On the other hand, logistics chains can also run through Arctic regions where temperatures can be below −10 °C [14]. In all of the above situations, it is essential to use microbiologically stable foods, as these foods can remain at temperatures below/above 25 °C for relatively long periods (weeks or months) during transport and storage [18].

From a nutritional point of view, it is advisable to incorporate dairy products into these food rations, especially as a potential source of essential nutrients needed for the physical and mental health of personnel. However, the range of dairy products that meet the above shelf-life requirements is very narrow and is practically limited to concentrated/condensed milk, powdered milk products and SPC. As a representative of a technologically non-acidic food, the only way to achieve the required shelf life of 24 months at ambient temperature for PC is to sterilize it, and thus, it is necessary to inactivate not only the vegetative forms of microorganisms (MO) but also their potential spores during the preservation process [19,20].

### 2.2. Production of Sterilized Processed Cheese

Production of SPC starts in the same way as for regular PC. The manufacture of PC by the conventional discontinuous process involves (i) the preparation of the mixture to be melted, (ii) the determination of the mixture of emulsifying salts and other additives, (iii) the actual melting process, (iv) high-pressure homogenization, if necessary, and (v) packaging, cooling and storage. The first step in production is the preparation of the raw material composition. The choice of natural cheese and other raw materials influences the final characteristics of the end-product and the formulation of the raw material composition is therefore very important. Obviously, only high-quality raw materials can be used in the manufacturing. Equally important is the choice of the composition of the emulsifying salts and their quantity (usually 2–3% *w*/*w*; calculated based on the total weight of the mass to be melted). The actual melting takes place in a processing equipment, into which all raw materials are fed and, after the equipment has been closed, the mixture is heated (indirectly through the intermediate layer or directly by steam injection, or by a combination of these) to a melting temperature (usually 90–110 °C) under reduced pressure and constant stirring, which is maintained for several minutes. In some cases (particularly for products with a high water and fat content), high-pressure homogenization is also included in the production process. The choice of process parameters has a significant effect on the properties of the PC produced. The manufactured product is hot-packed and stored at 4–8 °C after cooling. PC can be packaged in aluminum foils, plastic cups, tubes, casings, or jars. In the case of PC intended for sterilization, it is obviously necessary to choose a packaging suitable for this preservation procedure (usually aluminum tins with sealable lids) [21,22,23,24].

**Figure 1:**
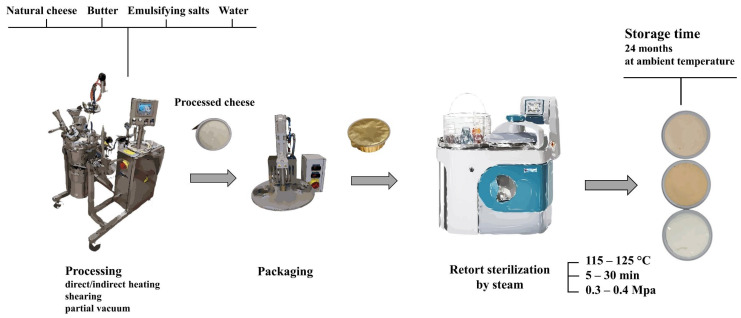
Schematic representation of the manufacturing steps applied in sterilized processed cheese (discontinuous process).

In addition to the traditional discontinuous process, PC (or in particular processed cheese sauces, dips, etc.) can also be made continuously. Continuous process usually uses sterilization temperatures of 130–145 °C for a few seconds. At these temperatures, bacterial spores are inactivated and, in the case of aseptic filling (which, however, is not usually performed in practice), the product would meet the conditions of commercial sterility (see Section 3). Another way of producing SPC for which commercial sterility is achieved is to sterilize the product in the packaging at 115–125 °C for several (tens of) minutes in batch sterilizers (retorts, autoclaves). In the retorts, an overpressure of about 0.3–0.4 MPa is maintained. In view of the normal pH of PC (5.6–6.0), temperatures equivalent to 121.1 °C with a minimum holding time of 10 min are necessary to ensure food safety. This sterilization regime is needed to inactivate bacterial spores (mainly *Bacillus*, *Geobacillus* and *Clostridium* genera) [2,8,25]. A scheme for the discontinuous production of SPC is shown in Figure 1.

## 3. Principles of Sterilization

A variety of preservation methods are used to extend the shelf life of foods, the main aim of which is to ensure their safety while preserving (as far as possible) the original quality of the food. Preservation treatments prevent chemical reactions in the food and the multiplication of MO. The intensity of food decomposition is directly proportional to the quantity and resistance of the MO and inversely proportional to the resistance of the food. Depending on whether the preservation method acts directly on the MO or increases the resistance of the environment, the methods are divided into abiotic and anabiotic. Abiosis is the direct inactivation of MO and the preserved food thus contains less MO than before the treatment. Indirect MO inactivation (or anabiosis) increases the resistance of the food by physical or physico-chemical treatment, after which the food becomes an unsuitable environment for the multiplication of MO. One of the most common abiotic method and, simultaneously, the most widely used thermal processing technology is sterilization [26,27].

Food treated with sterilization must meet the conditions of commercial sterility, which means the absence of viable MO that could multiply under circulating conditions and the absence of foodborne disease-causing MO. A level of microbial contamination is achieved that ensures safety and stability over the expected shelf life. The product is not sterile, but the MO present are in such numbers and forms that the product cannot be endangered. Absolute sterility is only theoretical and is neither necessary nor desirable for foodstuffs. Theoretically, it is not even achievable because the survival curve of MO crosses zero at infinity [28,29].

The course of sterilization is influenced by a number of factors, e.g., the properties of the MO, the composition of the food (content of antimicrobial or osmotically active substances, etc.), the humidity and acidity of the environment, the initial concentration of the MO, and last but not least, of course, the temperature and the duration of its effect. With regard to the acidity of the food, a distinction is made between so-called technologically acidic foods with pH < 4.0, in which spores of *Bacillus coagulans* and *Clostridium botulinum* cannot germinate, and so-called technologically non-acidic foods with pH > 4.0. In practice, this means that in foods with pH 4.0 and below, it is not necessary to inactivate bacterial spores by heating, as they cannot germinate and form toxins in an acidic environment. To assess the effect of heating, it is always necessary to consider the worst heated area of the packaging [30,31].

Sterilization heating can be characterized as an approach leading to the inactivation of vegetative forms of MO (bacteria, fungi, yeasts and viruses) including their spores. Inactivation of MO occurs after reaching an inactivation temperature that leads to the interruption of vital functions. In the case of technologically non-acidic foods, temperatures of 110–130 °C are used, with the duration of heating depending on the nature of the food. In general, however, the length of heating decreases with increasing sterilization temperature. Technologically acidic foods can be sterilized at lower temperatures, typically 70–100 °C [31,32]. In addition to inactivation of the microflora, undesirable enzymes that may adversely affect product properties and also microbial toxins are inactivated. *C. botulinum* is a bacterium that is generally considered to be the most hazardous thermotolerant sporulating MO, whose spores can germinate in inadequately sterilized product and the toxin produced is fatal. Some bacteria (*Geobacillus stearothermophilus* and *Thermoanaerobacterium thermosaccharolyticum*) are even more resistant to high temperature and are therefore used to prove commercial sterility [26,33]. It is important to note that thermophilic MO may be present in sterilized foods that are harmless under normal storage conditions and pose no risk to human health. If these foods were stored at tropical temperatures (above 35 °C), these MO could multiply and cause bombing or can rupture. Therefore, foods intended for storage in such conditions need to be sterilized under more restrictive regimes [31].

Spoilage of sterilized foods can occur as a result of non-microbial (enzymatic or chemical) and microbial changes. It is the result of either insufficient heating or inadequate cooling, or post-process contamination of non-hermetically sealed cans. Spoilage can be caused mainly by thermophilic aerobic and anaerobic sporulating bacteria, mesophilic sporulating bacteria, possibly also by some non-sporulating bacteria and less frequently by yeasts and molds. Spoilage resulting from chemical reactions may include hydrogen bombing caused by reactions of the food with the iron in the can, discoloration of the can or food, metallic flavor, turbidity of the brine or nutritional loss. Rancidity can also be caused by some thermotolerant bacterial enzymes, especially proteinases (bitter taste, gelation) and lipases (rancid taste) [28].

### 3.1. Types of Sterilization

**Figure 2:**
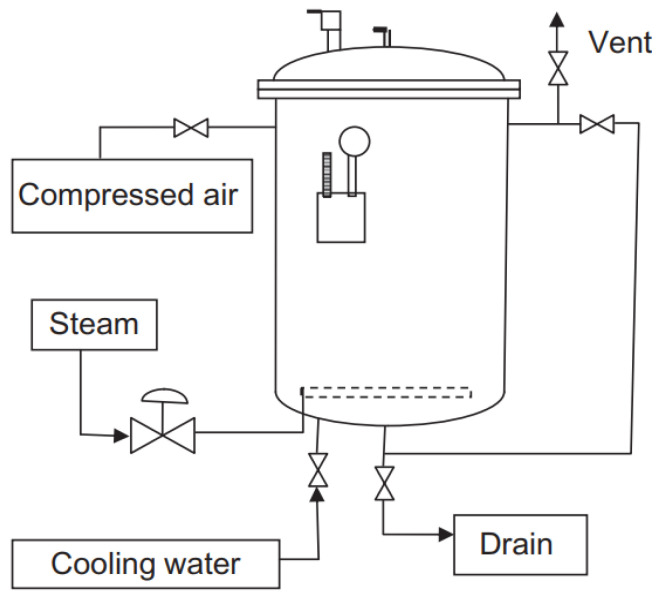
Simplified scheme of a vertical batch retort.

Sterilization can be performed in two basic ways—in the packaging or outside the packaging. In-container sterilization uses sterilizers (autoclaves, see Figure 2), i.e., pressure retorts that use steam as the heating medium. There are also continuous sterilizers, but the more common ones are still the classic batch (discontinuous) devices. More modern sterilizers are rotary, which speeds up heat transfer and prevents overheating. In-container sterilization is primarily used for solid foods. In the case of out-of-package sterilization, we speak of UHT (ultrahigh temperature) treatment, where the product is heated with superheated steam and then aseptically filled. This type of sterilization is preferably used for liquids [34,35,36,37]. When sterilizing in a container, it is necessary to ensure that the containers (cans, jars, etc.) are hermetically sealed. Such packaging is secure against MO penetration and preserves the commercial sterility of the contents even after the sterilization [38].

In the canning industry, metal cans are traditionally used, but less often we can also encounter glass or plastic versions. The most common material used for the production of metal cans is steel, a material characterized by excellent thermal conductivity, impermeability to light and oxygen, strength and the ability to protect food against external influences in the long term. Steel sheet with a thickness of 0.2–0.3 mm needs to be surface-treated to prevent interaction with food components (in particular migration of metal ions), which is usually achieved by tinning, chrome plating or nickel plating (0.5–2.5 μm) and possibly by covering with a layer of varnish. Aluminum with a sealable lid is also used for some cans. Glass cans are less commonly used because of their lower mechanical resistance. In addition, flexible pouches and semi-rigid polymeric trays have been an alternative to metal cans due to price increases and storage and transportation costs. Flexible packaging made of laminated foils composed of polypropylene, polyethylene, polyester or nylon, possibly with an aluminum layer, is nowadays available [39,40,41].

The sterilization process in a classic stationary vertical retort involves inserting the carrier with cans, closing the autoclave, filling the sterilizer with water so that the cans are submerged, supplying steam, heating the contents, actual sterilization (maintaining the prescribed temperature for a given period of time) and cooling with cold water. During cooling, the pressure in the retort must be controlled to prevent damage to the cans from internal overpressure. Either the autoclave temperature must be reduced slowly to prevent the overpressure in the cooling cans from reaching dangerous levels, but it is preferable to use a back-pressure sterilizer. During the cooling of containers, when a vacuum is created, MO can penetrate the imperfectly sealed packaging and cause contamination; therefore, the cooling water can be chlorinated or otherwise decontaminated [42]. Heat sharing is necessary for a successful autoclave sterilization process. There are three basic methods of heat sharing–conduction, convection and radiation. Heat transfer in sterilization involves heat transfer from a higher temperature environment (hot water, steam) to the surface of the container, conduction of heat through the packaging material, and heat transfer from the container to the interior of the food [36,43].

### 3.2. Microorganism Inactivation Kinetics

The amount of heat required to inactivate the MO is an important characteristic that must be known in order to determine the appropriate sterilization regime (given by the combination of the sterilization temperature and the time over which this temperature is applied) needed to ensure a safe and stable product. Traditional canning technologies use two factors, the *D*-value and the *z*-value, to determine this mode. The decimal reduction time (*D*-value), is defined as the time it takes to inactivate 90% of the vegetative forms of MO and their spores (see Equation (1)). It is given in minutes at the temperature to which it refers.

(1) $$\log N=logN_{0}-\frac{t}{D}$$

where *N* is the amount of surviving MO, *N*_0_ is the initial amount of MO, *t* is the time in minutes, and *D* is the decimal reduction time in minutes [44].

**Figure 3:**
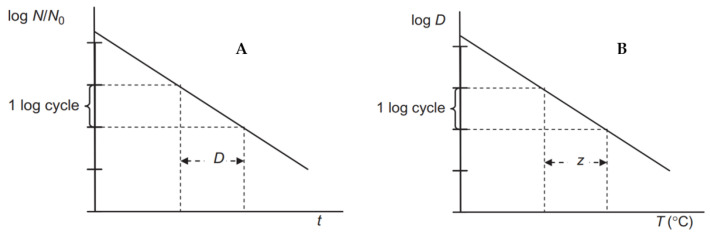
The log-linear model of thermal destruction of microorganisms (part **A**) and the dependence of *D*-value on temperature (part **B**).

A reduction of 90% in the number of MO represents a reduction of one order of magnitude in the number of MO. If we consider the MO lethal curve as a linear dependence of the logarithm of the concentration, i.e., the number of orders of magnitude, on time, then each 90% reduction in the number of MO is achieved in the same time of exposure to the relevant temperature (see Figure 3, part A). The decimal reduction time depends on the heating temperature. As the heating temperature increases, the time required to reduce the number of MO by one order of magnitude becomes shorter. *D*-values are used to assess the thermoresistance of individual MO and are also used to assess the inactivation effect of sterilizing heating. They are also used in optimizing the heating process, i.e., determining the process that will lead to the desired inactivation of MO while ensuring minimal quality changes in the food [45].

The temperature dependence of the *D*-value, the so-called *D*-*T* thermoinactivation curves, were empirically determined for each significant MO. They can be reported either as the dependence of the heating temperature on the heating time, *T* = f (*D*), or as the dependence of the time on the temperature, *D* = f (*T*). From these inactivation curves, a curve slope, i.e., the *z*-value (°C), referred to as temperature sensitivity, can be determined [46].

The *z*-value expresses the change in the *D*-value with temperature (see Equation (2) and Figure 3, part B). Size of the *z*-value is given by the increase in temperature that is required to reduce *D*-value by 90% (or one logarithmic order of magnitude). In other words, it is the number of degrees Celsius by which the temperature must be increased to produce a tenfold reduction in the *D*-value.

(2) $$logD_{T}=logD_{ref}-\frac{T-T_{ref}}{z}$$

where *D_T_* is the *D*-value in minutes at temperature *T* and *D_ref_* is the corresponding value at the reference temperature *T_ref_* (usually 121.1 °C).

The thermoinactivation curves are not usually shown graphically, but the *D*-values and *z*-values for each MO are tabularized for a particular temperature. Similarly, the values expressing the progression of destruction of enzymes and other important food components are tabularized (see below) [47].

### 3.3. Setting Sterilization Limits

A measure of the inactivation effect of the sterilization process can be obtained by using the ratio *D/D_T_*. The lethal ratio *L* expresses the inactivation effect of any temperature *T* on a MO of temperature sensitivity *z*. It is defined as “one minute of heating to temperature *T* has the same inactivation effect as *L* minutes of heating to the reference temperature *T_ref_*”. It can be calculated from Equation (3).

(3) $$L=10^{\frac{T-T_{ref}}{z}}$$

The lethal ratio for temperatures higher than *T_ref_* will be greater than 1, and for temperatures lower than *T_ref_* it will be less than 1. For example, for *T* = 111.1 °C and *T_ref_* = 121.1 °C is *L* = 0.1 min, which means that 1 min at 111.1 °C is equivalent to 0.1 min at 121.1 °C. Since the temperature at the slowest heating point of the container varies with time, it is necessary to determine the lethal effect of each temperature. The sum of the lethal ratios obtained at each temperature per time unit is known as the inactivating (sterilizing) effect, the so-called *F*-value (see Equation (4)) [48].

(4) $$F=\int_{0}^{t}10^{\frac{T-T_{ref}}{z}}dt$$

The inactivating effect of a particular treatment is not expressed as a decrease in the amount of MO but is mediated by the effect that warming the food to *T_ref_* temperature for *F* minutes has. When using Equations (3) and (4), it is necessary to know the *z*-values of the target MO. For non-acidic foods, a *z*-value of 10 °C corresponding to the value usually used for *C. botulinum* is used. In some cases, spores of the thermophilic bacteria *C. thermosaccharolyticum* and *G. stearothermophilus* are also considered. Furthermore, the *F*_0_-value expresses the *F*-value for a reference temperature of 121.1 °C and a *z*-value of 10 °C [31,35].

Based on the value of *z* = 10 °C for *C. botulinum*, it was determined that any thermal heating that reaches the threshold of *F*_0_ = 3 min is considered sufficient to inactivate pathogenic MO. The second approach, which is considered traditional in canning, is the 12-*D* concept, i.e., reducing the bacterial population by 12 logarithmic orders (e.g., from 10^6^ to 10^−6^). Both of these approaches ensure the practical sterility of the canned food [49,50].

To determine the heat transfer through the container, it is necessary to know the temperature-time profile at the slowest heating point of the container. This is obtained experimentally by placing a temperature sensing device (called a datalogger) in a suitable location in the packaging. The most commonly used thermocouples are made of copper and constantan (a 55:45 copper-nickel alloy whose resistivity is approximately constant over a wide range of temperatures). In addition to thermocouples, resistance thermometers can also be used. Heat transfer is influenced by 3 types of factors. The first factor is related to the sterilization process itself, i.e., the temperature and duration of sterilization, the temperature medium used, and possibly mixing. The second group of factors relates to the product being sterilized and includes consistency, initial temperature and microbial load, pH or presence of additives. Finally, the third group is the factors connected with packaging, especially material and shape [51,52].

### 3.4. Setting Sterilization Parameters to Maximize Product Quality

Thermal heating during sterilization does not only affect the MO, but of course also the food itself. The available literature generally indicates that to minimize qualitative changes in sterilized foods, it is recommended to use the highest temperature applied for the shortest possible time. Therefore, the limiting factor of sterilization is the rate of heating and cooling of the product. The severity of the heat exposure depends primarily on the nature of the food. While foods in which heat is distributed by convection (mainly liquids–beverages, homogeneous soups, etc.) are heated relatively quickly and evenly, foods in which conduction predominates (especially in the case of in-container sterilization of solid foods) are heated unevenly; the edges of the container are heated more than the center. There are several options to reduce the effects of thermal heating, such as changing the packaging, using a higher temperature acting for a shorter time, using less intense heating followed by cold storage, acidifying the product followed by pasteurization, or using microwave or resistance heating [45,53].

While the *z*-value expressing the effect of temperature on nutritional and organoleptic parameters is in the range of 15–50 °C, for MO destruction it is 4–12 °C; the literature usually indicates that the *z*-values for microorganisms are 3–10 times lower. In general, a 10 °C increase in temperature doubles the sterilizing effect, while microbial inactivation increases tenfold. It is therefore true that the same inactivation effect with less quality change can be achieved by increasing the treatment temperature for an order of magnitude shorter time. The effect of temperature on the quality parameters is quantified by the *C*-value defined in Equation (5).

(5) $$C=\int_{0}^{t}10^{\frac{T-T_{ref}}{z_{c}}}dt$$

where *z_c_* is *z*-value for quality parameters.

As can be seen from Equations (4) and (5), the *C*-value is equivalent to the *F*-value but is based on kinetic parameters of nutrients and other chemicals instead of MO; it is also referred to as the chemical change index [34,45,53].

## 4. The Effect of Sterilization and Long-Term Storage on the Quality of Sterilized Processed Cheese

Preservation methods that use high temperatures to preserve food result in nutritional losses, changes in texture and color, and the development of foreign tastes and odors [35]. Since the early 19th century, when Nicolas Appert discovered thermal heating as a method of food preservation, there has been a tendency to mitigate quality losses during heat treatment. In canning industry, there are two main requirements–ensuring health safety and maintaining the quality parameters of the fresh product. Hence, the aim is to optimize these two conflicting requirements [31]. The main reactions that result from sterilization include MRC and LOR. Markers of these reactions include ammonia and secondary lipid oxidation products [often expressed as thiobarbituric acid reacting substances (TBARS) values). Increases in these markers are associated with changes in other food properties–in particular color, consistency, taste and flavor, and therefore sensory quality. Higher carbohydrate, protein and fat content in food results in higher levels of MRC precursors (amino acids and reducing sugars) and LOR precursors (fatty acids) [54,55].

Despite the fact that sterilized foods can be considered non-perishable, they are not fully stable during long-term storage. Hence, these foods can be expected to develop physico-chemical changes. These changes will occur both in the macronutrients (i.e., carbohydrates, proteins and lipids) and in the micronutrients present (vitamins, minerals, etc.) [13]. The manufacturer usually recommends that long-life foods should be stored at normal ambient temperature, usually below 25 °C. At this temperature, quality changes can be expected to be more significant than at refrigeration temperature. During transport of food in humanitarian missions and emergencies, these foods may spend a considerable time at sub-zero temperatures (in Arctic regions) or well above the recommended storage temperature (in subtropical and tropical regions). At these temperatures, changes in nutritional parameters, deterioration in sensory quality and, as a consequence, a reduction in shelf life could be expected. It is therefore desirable that food spends as little time as possible at these extreme temperatures.

### 4.1. Protein Changes

The main protein reactions that occur due to the high temperature used in food preservation are undoubtedly MRC. It is a complex of reactions between amino compounds (amino acids, peptides, proteins, biogenic amines) and carbonyl compounds (mainly reducing sugars). In the course of these reactions, highly reactive carbonyl compounds are formed which react with each other and with the amino compounds present. The products of MRC are brown pigments, melanoidins, whose chemical structure remains unclear because melanoidins are complex and heterogeneous polymers. On the other hand, also various low-molecular-weight pigments formed by the MRC have been reported and are supposed to be melanoidins precursors [56]. MRC is one of the non-enzymatic browning reactions. Moreover, non-enzymatic browning reactions include degradation of ascorbic acid, lipid peroxidation and caramelization [57].

**Figure 4:**
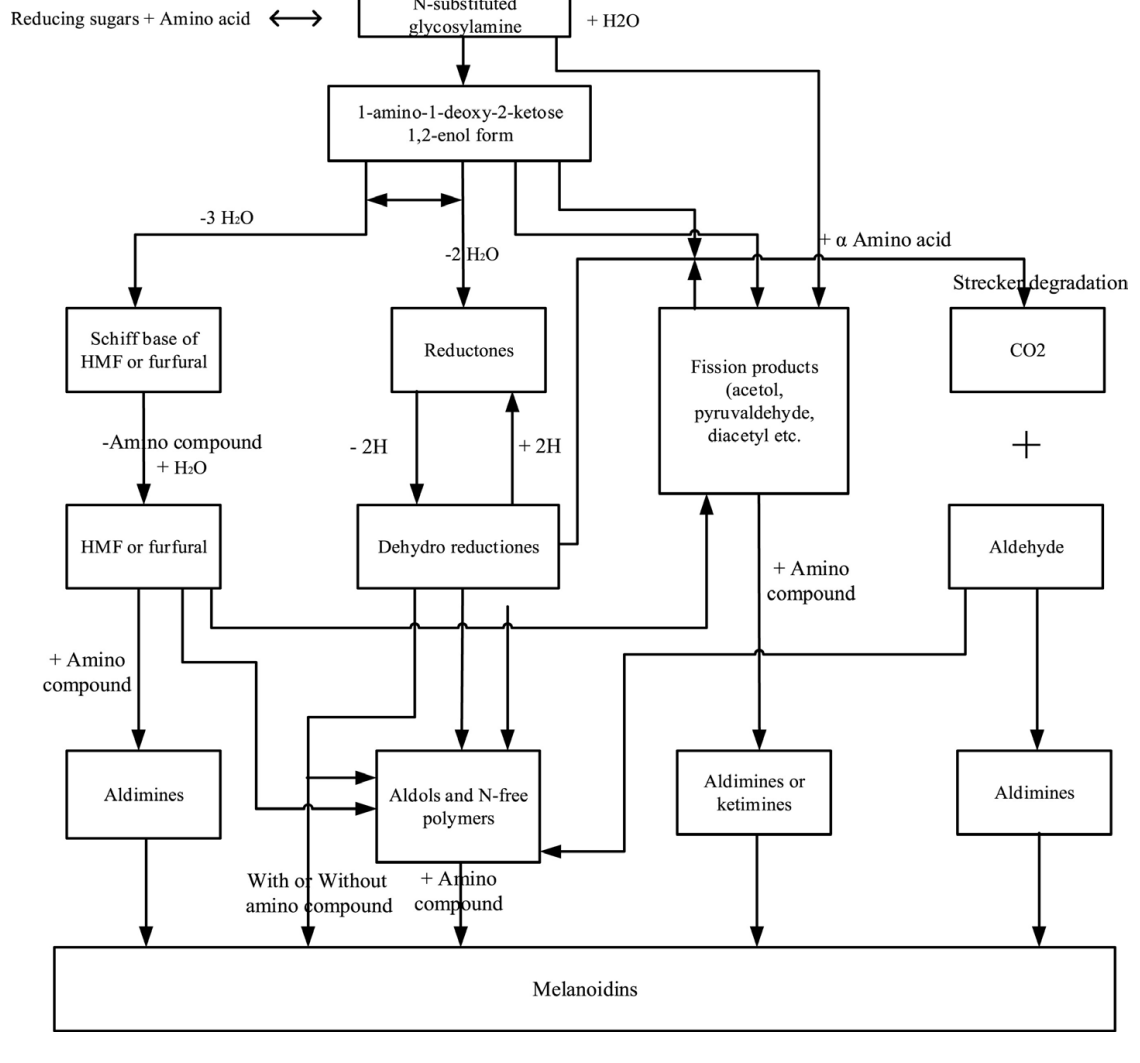
Overview of Maillard reaction complex scheme [62].

Due to the complexity of MRC, it is still impossible to present a complete reaction scheme and only a small fraction of the resulting compounds have been characterized. Simplistically, MRC can be divided into three phases:

- the initial phase represents the formation of glycosylamine followed by the Amadori shift (in the case of an aldose reaction) or the Heyns shift (in the case of a ketose reaction);
- the intermediate phase involves dehydration and fragmentation of carbohydrates and Strecker degradation of amino acids;
- the final/advanced phase represents the reactions of intermediates that lead to the formation of heterocyclic compounds (usually fragrances and flavors) and high molecular weight melanoidins [58,59,60]. A scheme of the MRC is shown in Figure 4.

The main factors that influence the progress of MRC and that can be used to control the progress of these reactions in food processing include temperature, reaction duration, pH of the medium, water activity and the type and availability of reactants. The reaction can be inhibited by removing one of the reaction partners, adjusting the water content, lowering the temperature, reducing the heating time, adjusting the pH or adding substances that slow or inhibit the reaction [61].

MRC impairs the nutritional value of foods, primarily due to amino acid (AA) degradation, loss of vitamins, impaired digestibility and reduced availability of iron, phosphorus and magnesium. Protein functionality (heat stability, solubility, emulsifying properties, foaming, gelation, textural properties) is also affected by MRC. Other consequences of MRC are organoleptical properties (color, flavor and texture) deterioration [57,58,63,64]. Ammonia is an important indicator of MRC, which is produced in many reactions and its content is closely related to the amount of AA [65,66]. The increase in ammonia content in PC due to sterilizing heating was described in studies [8,9,19].

Buňka et al. [55] studied the effect of sterilization on AA content in PC and described a decrease in cysteine, methionine, leucine, arginine, aspartic and glutamic acids, serine, alanine, lysine, histidine and isoleucine. In contrast, the levels of threonine, proline, tyrosine, valine and phenylalanine were not affected. The degradative changes in AA included deamination reactions that caused an increase in ammonia. Lazárková et al. [20] dealt with the effect of 4 different sterilization regimes (given by the combination of temperature and time) on the quality of PC. The application of sterilization regimes 110 °C/100 min and 115 °C/32 min caused a decrease in AA content by an average of 10% (compared to non-sterilized PC); the most sensitive AA were cysteine, methionine, serine, threonine and tyrosine. The amount of AA decreased by about 5% for the regime 120 °C/10 min and by only 3% for the regime 125 °C/3.2 min.

However, during sterilization, not only the direct destruction of AA may occur, but also the formation of bonds that are difficult to cleave in the human digestive tract. A typical AA involved in the formation of these bonds is lysine. The consequence is a lower utilization of lysine [67]. However, in standard analysis of AA content, this unavailable lysine is included in the total determined lysine content, thus overestimating its amount from a nutritional point of view. Since lysine is an essential AA that is often considered as a measure of the biological value of proteins, it is essential to know the content of available lysine [68]. There are derivatization methods that allow the analysis of only the available lysine. One of the most widely used is the Carpenter method (derivatization with 1-fluoro-2,4-dinitrobenzene). Knowing the content of available and blocked (unavailable) lysine facilitates the evaluation of the effect of thermal heating on protein quality [60]. Lazárková et al. [19] reported a decrease in lysine content in PC as a consequence of sterilization. While in non-sterilized PC, the proportion of available lysine was 98%, the sterilization resulted in a decrease of up to 2% in regimes 115 °C/32 min, 120 °C/10 min and 125 °C/3.2 min, while in regime 110 °C/100 min the decrease in the amount of available lysine was 3–4% (with lactose content up to 1% *w*/*w*), or 6–10% in the case of higher lactose concentration (up to 2% *w*/*w*).

The publication by Gaucheron et al. [69] monitored the proteolysis of proteins during milk sterilization, based on the increase in non-protein nitrogen content and the release of low molecular weight peptides from casein micelles. These reactions, together with other protein changes (e.g., dephosphorylation, deamination, lysinoalanine formation, formation of disulfide bridges) may influence the content of AA in the food. Lazárková et al. [20] analyzed the protein profile of PC by sodium dodecyl sulfate polyacrylamide gel electrophoresis (SDS-PAGE) and evaluated the profiles by cluster analysis. The dendrogram obtained showed that non-sterilized PC differed significantly from sterilized samples and that sterilization caused cleavage of proteins with molecular weights above 20 kDa. The protein profile of PC was influenced both by the applied sterilization regime and by the amount of lactose present. More significant hydrolytic changes in proteins were observed with decreasing sterilization temperature (and longer exposure time) and increasing lactose content.

According to Daly et al. [70], PC are susceptible to browning and color changes caused by MRC, particularly due to the high temperatures used in their production, as well as their higher pH (>5.9). Buňka et al. [71] described PC darkening as a consequence of sterilization and higher storage temperatures. Fann et al. [72] observed browning of PC during storage and suggested vitamin E, citric acid and cysteine as inhibitors of this process. Darkening of PC as a consequence of sterilization has also been described in the literature [8,9,19,20]. As a consequence of sterilization, most PC showed a shift in chromaticity to the yellow and red region. In the case of regimes 110 °C/100 min and 115 °C/32 min, there was also a decrease in L* (brightness/luminosity). Increasing lactose content in the sterilized PC led to a decrease in L* and an increase in a* (chromaticity on the green-red axis) and b* (chromaticity on the blue-yellow axis). For samples sterilized with regimes 120 °C/10 min and 125 °C/3.2 min, this trend was only evident at lactose contents ≥1% *w*/*w* [20]. These trends were confirmed by sensory analysis of SPC color in the study of Lazárková et al. [19]. Furthermore, Jedounková et al. [9] and Buňka et al. [8] also reported that SPC were darker, redder and yellower compared to non-sterilized PC samples. Song et al. [73] concluded excessive color changes in cream cheese treated with both UHT sterilization (137 °C/4 s) and autoclave sterilization (121 °C/10 min). According to Ranvir et al. [74] UHT treatment resulted in an increase in color value of milk. Additionally, Balde and Aider [75] determined increased b* values after sterilization of concentrated skim milk.

The increase in hardness due to sterilization can be explained by casein aggregation, which results in a multiplication of interactions between proteins in the casein network. During MRC, new bonds are formed between proteins (e.g., isopeptide bonds via the ε-amino group of lysine), which further contributes to the increasing intensity of protein binding [11,66,76]. The hardening of PC due to sterilization and also during 24 months of storage was described by Buňka et al. [71]. Jedounková et al. [9] reported the increase in hardness due to sterilization by both textural profile analysis (increase in force required to compress the sample) and rheological analysis (increase in complex modulus of elasticity G*). Buňka et al. [8] stated similar conclusions, i.e., rising of the hardness and storage (*G*′) and loss (*G*″) moduli in SPC compared to the non-sterilized PC. According to Li et al. [77] SPC (121 °C/15 min) stored at 25 °C exhibited higher hardness values compared to pasteurized PC (100 °C/15 min) stored at 4 °C.

Long et al. [78] subjected samples of whipping cream to different sterilization conditions (boiling sterilization, autoclaving and UHT sterilization). Authors concluded that higher sterilization intensity led to a denser network structure causing higher viscosity and more solid-like structure of whipping cream. Heat treatment induced interactions among milk proteins and fat globules. Similarly, according to Wang et al. [79] increasing sterilization intensity caused more extensive thickening (i.e., higher viscosity) of restored dairy cream subjected to different sterilization regimes. Furthermore, Dhungana et al. [80] declared that sterilization of cream significantly increases the MRC between protein and lactose, leading to impairment of the cream stability since very reactive intermediate products are formed during the reaction. Heat-induced interactions of proteins and aggregation and coagulation of fat globules of the infant formula emulsions were described by McSweeney et al. [81]. Decreasing stability of cream cheese (due to fat globule aggregation) as a result of sterilization was concluded by Song et al. [73]. Slight increase in viscosity of milk after UHT treatment was indicated by Ranvir et al. [74]. Lowering of heat stability (casein coagulation, sediment formation) as a result of both in-container and UHT sterilization of concentrated milk was noted by Dumpler et al. [82]. Moreover, Balde and Aider [75] stated that the apparent viscosity of concentrated skim milk increased during sterilization treatment (due to increase in casein micelle size by the denatured whey proteins).

MRC and LOR lead to off-flavors and off-odors, as they produce large amounts of organoleptically active compounds, causing deterioration of the flavor of sterilized foods. Many of these compounds (such as acrylamide, heterocyclic amines, furan, hydroxymethylfurfural, carboxymethyllysine) can have negative effects on human health, being toxic and/or carcinogenic [11,57,58]. Valero et al. [83] studied the changes in volatile component of milk after UHT treatment. Increase in methyl ketones, dimethyl sulfide, dimethyl disulfide, and aldehydes was concluded. Li et al. [77] concluded that sterilization of PC resulted in higher levels of hydroxymethylfurfural (one of the major products of MRC that is used to indicate the degree of heat treatment) and dicarbonyl compounds (glyoxal and methylglyoxal, highly reactive intermediates in MRC). Bertrand et al. [84] heated PC to temperatures of 80–150 °C and monitored the evolution of volatile compounds, focusing on the fraction of aromatic compounds that may contribute to the sensory characteristics of PC. The markers of LOR, caramelization and MRC were of interest. Maltol and furaneol, which are formed by degradation of lactulosyllysine, were identified as the main volatiles responsible for the “burnt” odor. The limiting temperature above which this defect developed significantly was set at 120 °C. In their work, the authors recommended (to minimize the formation of off-odors) that food should spend as little time as possible at temperatures above 120 °C and that as little lactose as possible should be included in the raw material composition. A maximum lactose addition of 1% *w*/*w* (to avoid undesirable organoleptic changes) to PC was also recommended in publications Lazárková et al. [19] and Lazárková et al. [20]. Authors observed more significant changes in the sensory quality of PC, especially flavor, with decreasing sterilization temperature (and longer exposure time) and, simultaneously, increasing lactose content. While the non-sterilized samples and the PC sterilized using the regime of 125 °C/3.2 min were rated as very good or good, the PC sterilized using the regimes of 110 °C/100 min and 115 °C/32 min were, especially with increasing lactose content, less good or even unacceptable. Deterioration of flavor of PC as a result of sterilization was also observed by Jedounková et al. [9] and Buňka et al. [8]. Increasing concentrations of MRC by-products (e.g., furosine), higher fluorescence intensity and growing levels of volatile compounds (aldehydes, ketones, sulfides) leading to sensory quality deterioration were also reported by Song et al. [73].

MRC can also lead to the formation of process contaminants that can be carcinogenic and mutagenic (in particular heterocyclic aromatic amines, e.g., pyridoimidazoles, pyridoindoles and tetraazafluoranthenes). The course of MRC is influenced by the raw material composition (i.e., different protein, lipid and carbohydrate contents), water content and water activity, the method of heat treatment (temperature and its duration), the course of the temperature gradient during heating and cooling, etc., [65,66,85].

Other reactions of proteins and AA caused by sterilization include, e.g., (i) the reaction of bound asparagine and glutamine with free/bound lysine, during which ammonia is released and an isopeptide bond is formed that is not digested by enzymes in the human digestive tract, (ii) the release of sulfate from bound cystine, and (iii) Strecker degradation (oxidative decarboxylation) of AA leading to the formation of carbon dioxide, ammonia and aldehydes [69,76,86].

During food storage, similar reactions to that occurring during sterilization can be expected. The extent of non-enzymatic browning during storage increases with increasing levels of non-reducing carbohydrates, especially lactose, with increasing storage temperature and with the progressive formation of oxidized lipids [7,87]. Various protein changes have been described during storage of UHT milk, in particular proteolysis, protein crosslinking, color changes and formation of volatiles [83]. Color changes in UHT milk during 90 days storage at ambient temperature were studied by Popov-Raljić et al. [88] who suggested that chroma (b*) increase serves as an indicator of defects or damages occurring during storage, especially with respect to nonenzymatic milk spoilage. Zeren et al. [89] observed decrease in pH, increase in viscosity and darkening of UHT milk stored for 5 months at 25 and 41.5 °C. Karlsson et al. [90] concluded a decreased shelf-life of UHT milk stored at 30 and 37 °C compared to that stored at 4 and 20 °C. Authors reported sediment formation and taste and color impairment. Sedimentation in UHT milk was also observed by Malmgren et al. [91]. While sedimentation was noted after 6 months for samples stored at 5 °C, it was present after 3 months for samples stored at 30 and 40 °C. Ranvir et al. [74] analyzed increase in viscosity, a* value, b* value, hydroxymethylfurfural and lactulose content and, furthermore, decrease in pH and L* value during the storage of UHT milk. Sedimentation content growth and Maillard browning negatively affected the quality of UHT milk. Furthermore, Al-Saadi and Deeth [92] studied changes in UHT milk during storage at different temperatures. Authors observed a greater decrease in pH and increase in browning and proteolysis in samples stored at 37 and 45 °C than in samples stored at 5 and 20 °C; these changes were positively related to both time and temperature of storage. Protein profiles of samples kept at 45 °C were completely different from that of a sample stored at 5 °C. Hence, considerable changes took place in the proteins of samples stored at elevated temperature. Additionally, the above-mentioned authors described an increase in lysinoalanine concentration; an indicator of covalent, non-disulfide cross-linking of proteins. The cross-linking was also observed by SDS-PAGE.

A decrease in the amount of total AA and the increase in ammonia content during long-term storage of PC been reported by Bubelová et al. [12]. While the total amount of AA decreased by 2% after one year and by 4.5% after 2 years during storage in the fridge (6 °C), the decrease for samples kept in thermostat (40 °C) was 7.5% after one year and almost 12% after 2 years. The essential amino acid index values decreased by 4, 6 and 12% for samples stored at 6, 23 and 40 °C, respectively. The observed decrease in AA content corresponded with the increasing amount of ammonia as their degradation product. The extent of proteolysis in this study was monitored by SDS-PAGE. The dendrogram showed that the protein profile of samples stored for 24 months in the refrigerator and at room temperature was similar (17 and 13 proteins with molecular weights ranging from 3.7 to 28.2 kDa were detected in these samples, respectively). Samples stored in the thermostat formed a separate cluster and only 5 proteins with molecular weights of 9.6–28.6 kDa were detected. Furthermore, authors observed a deterioration in sensory quality associated, among others, with MRC. While the PC stored in the fridge and at 23 °C were evaluated as “good” or “less good” after two years, the samples stored in the thermostat were already “unsatisfactory” after 6 months and even “unacceptable” after 12 months. Additionally, PC stored at higher temperature were always marked as darker and less preferred in the paired tests [12].

Theoretically, enzymatic reactions can also be observed during storage of sterilized foods; Turner and Vulfson [93], Haki and Rakshit [94] and Synowiecki et al. [95], for example, have found that some thermostable enzymes retain their activity even at temperatures of 100–200 °C for several seconds. These enzymes usually require very low water content for their activity and are resistant to a wide range of pH.

Compounds of lipid nature, in particular primary and secondary products of LOR or free hydroxyl groups of partially or fully hydrolyzed triacylglycerols, may also be involved in protein interactions. Changes in proteins and lipids are thus often associated with each other in foods.

### 4.2. Lipid Changes

Among the most important reactions in which lipids participate due to high temperatures and during storage are oxidation reactions. Lipids can be oxidized in three main ways, auto-oxidation, enzyme-catalyzed oxidation and photo-oxidation. The most common type of oxidation in foods are auto-oxidation reactions. While at normal temperatures only unsaturated fatty acids (FA) are oxidized by air oxygen, at higher temperatures saturated FA are also auto-oxidized. Auto-oxidation is a radical chain reaction that takes place in three stages. The first stage (initiation) is the formation of a free hydrogen radical and a free FA radical. In the propagation stage, the peroxyl radical and subsequently hydroperoxides (the primary products of autooxidation) are formed. The final stage (termination) represents the reaction of the two radicals to form a stable non-radical product [96,97]. Secondary products of autooxidation include (i) compounds with the same number of carbons (cyclic peroxides, endoperoxides, epoxy acids, hydroxy acids and oxo acids), (ii) compounds with a lower number of carbons (volatile and sensory active aldehydes, ketones and hydrocarbons) and (iii) compounds with a higher number of carbons (polymer products). Factors influencing lipid oxidation include food composition (FA profile, fat, cholesterol, pro-oxidant and antioxidant content) and production and storage conditions (temperature used during production and storage, length of storage, access to light and oxygen, water activity) [98]. Oxidation of lipids can lead to the formation of trans-unsaturated fatty acids, which should be kept to a minimum in foods given the direct link to heart disease [99].

The initial stages of lipid oxidation in foods are usually monitored by HPLC analysis of hydroperoxides or by determination of the peroxide value. A measure of secondary oxidation products is usually the TBARS value, which expresses the amount of malondialdehyde and reflects the degree of lipid oxidation [54,87]. Increases in TBARS values due to sterilization of PC have been described by Jedounková et al. [9] and Buňka et al. [8].

According to Ajmal et al. [100], exposure of milk and dairy products to sterilizing temperatures and subsequent storage at room temperature leads to many biochemical changes, especially proteolysis and lipolysis. Lipid oxidation is the main reason for spoilage of these products. During sterilization and storage, there is an increase in free FA content, change in triacylglycerol profile, decrease in pH due to organic acid formation and deterioration in sensory quality. The authors monitored the progress of lipid oxidation on the basis of peroxide value, anisidine value and the amount of free FA and conjugated dienes, noting an increase in all parameters both due to UHT heating and during 90 days of storage. Li et al. [101] analyzed the changes in the oxidation stability of milk after different heat treatment and concluded that both the concentration of oxidized flavor compounds and TBARS value amplified with increasing intensity of heat treatment. Kristensen et al. [87] investigated the effect of light access and storage temperature (5, 20 and 37 °C) on the color and oxidative stability of PC. They concluded that higher storage temperature (37 °C) induced non-enzymatic browning and lipid oxidation, whereas light access caused only LOR. The product of the linoleic acid oxidation reactions may be conjugated linoleic acid, which has been detected in PC and has antioxidant, anticarcinogenic and antiatherosclerotic effects [102].

Sterilization also affects the size and shape of the fat globules in PC. Tremlová et al. [103] reported a reduction in the amount of small fat globules and, conversely, an increase in the number of large fat globules above 500 μm^2^ and, therefore, an increase in their surface area. Authors assumed that during heat treatment of PC the small particles probably merged, thereby increasing their area. The increase in fat globule size due to higher storage temperature has also been described in Bubelová et al. [12]. Furthermore, Li et al. [77] concluded fat globule changes due to aggregation of fat in sterilized processed cheese. Long et al. [78] and Wang et al. [79] stated that higher sterilization intensity resulted in the formation of larger fat globules (due to their coalescence), which caused an increase in viscosity of whipping cream and restored dairy cream, respectively. Similarly, Dhungana et al. [80] reported increase in fat globule size, clustering and eventually phase separation in non-homogenized sterilized cream. Changes in size distribution of milk fat globules were noted also by Li et al. [101] in heat treated milk.

The impact of heat treatment on lipid oxidation can also be observed via antioxidant capacity determination. Dias et al. [104] concluded reduction in total antioxidant capacity as a consequence of UHT treatment of milk. Additionally, free radicals produced during lipid oxidation can also attack proteins and cause oxidation and aggregation of proteins, leading to impaired digestibility and reduced availability. Oxidation of proteins is initiated by metal-, enzyme-, or light-induced processes and results in a loss of thiol groups, formation of protein carbonyls and specific oxidation products and a decrease in sensory quality (mainly due to changes in color and texture). The mechanism of oxidation reactions of proteins can be divided into three phases, similar to the auto-oxidation mechanism of lipids. In the initial phase, free radicals and hydroperoxides are formed. Subsequently, the radicals are transferred to peptides and proteins and during the final, termination phase, non-radical products are formed [59,97]. Poojary and Lund [59] stated that dairy products contain substantial levels of transition metal ions and riboflavin and are exposed to heat and light during processing and storage. Hence, dairy proteins may undergo reactive oxygen species-, enzyme (lactoperoxidase)-, and light-mediated oxidation.

### 4.3. Saccharide Changes

The most important reactions involved in the heating of food containing carbohydrates, i.e., MRC, were characterized in Section 3.1. At temperatures above 120 °C (but usually more typically at 150–190 °C), caramelization of carbohydrates takes place, during which brown to brown-black amorphous products of various compositions are formed. These reactions occur together with MRC and thus also contribute to non-enzymatic browning. The reactions, as well as the caramelization products, are similar to those described for MRC [105].

In UHT milk and sterilized dairy products, lactose is isomerized to form lactulose. Its content correlates with negative organoleptic characteristics (boiling flavor and darker color) in these products and is therefore used to evaluate heat treatment severity [106]. Increase in lactulose content after UHT treatment of milk was observed by Ranvir et al. [74] and Valero et al. [83]. Lactose can also degrade to form organic acids (formic, acetic or pyruvic), leading to an increase in titratable acidity of UHT milk during storage [100]. Decrease in pH of UHT milk was also noted by Ranvir et al. [74], both as a result of heat treatment and storage. Schär and Bosset [7] reported in their study that crystals of lactose and other compounds (mainly emulsifying salts, AA or calcium phosphate) can form during PC storage.

### 4.4. Vitamin and Mineral Changes

The content of most vitamins decreases during sterilization of various food. This reduction in content may not only be due to direct destruction of the vitamins but may also be due to irreversible binding of the vitamins to other food components (e.g., proteins) or conversion to less potent or inactive products [107,108]. Various authors reported significant losses of vitamin B_1_ [109], B_6_ [108,109], B_9_ [109], vitamin C [13] and vitamin A [110]. On the other hand, Sharma and Lal [109] concluded that vitamin B_2_ was relatively stable during various heat processing treatments of milk. Similarly, Kaushik et al. [111] reported insignificant losses of vitamin D_2_ during milk sterilization. The most abundant mineral in dairy products is calcium. According to Seiquer et al. [112] heat treatment reduces calcium solubility and absorption and, furthermore, impairs dietary calcium utilization. Overview of consequences of both sterilization and long-term storage on quality of sterilized dairy foods is given in Table 1.

## 5. Final Consideration and Recommendations

Sterilized processed cheeses are a promising group of dairy products, which, thanks to their extended shelf life, are used both in the regular market network and especially in the provision of meals in crisis situations. Although sterilization ensures a long shelf-life at ambient temperature, exposure to high temperature is associated with a number of qualitative changes affecting all the basic components of processed cheese (i.e., protein, fat and lactose). Moreover, even sterilized foods are not entirely stable and further quality changes can be expected during storage. This review focused specifically on the changes that occur due to the effect of sterilization temperature on processed cheese and also due to the effect of long-term storage. In particular, the complex of Maillard reactions and lipid oxidation reactions, which occur both during sterilization heating and during storage (especially at elevated temperatures) and which lead, for example, to a deterioration of nutritional parameters, reduced digestibility or impaired sensory quality, are discussed in detail. On the basis of a review of available scientific articles, the following recommendations can be proposed:

- It has been verified that a lower sterilization temperature applied for an adequately longer period of time causes more significant changes in quality and can lead, in extreme cases, to unacceptable products. For the production of sterilized processed cheese, the highest sterilization temperature for the shortest possible time is recommended.
- The high lactose content (mainly due to the inclusion of whey powder or milk powder in the raw material composition of processed cheeses to reduce production costs) leads to a more intense Maillard reaction complex. This results in more pronounced color changes (darkening) and the development of foreign tastes and odors. A limiting value for the lactose content to obtain acceptable products, 1% *w*/*w*, can be suggested.
- Higher storage temperature and longer storage time lead to more significant changes in the quality of sterilized processed cheese, mainly due to more intense Maillard reaction complex and lipid oxidation reactions, which is reflected in a deterioration of both nutritional and sensory quality. Therefore, in order to maintain the highest possible quality, appropriate storage conditions (especially refrigerator temperatures or as shortest time at elevated temperatures as possible) should be considered.

The possibility of masking the negative effects of sterilization heating (i.e., especially the darker color, off-flavor and off-odor) with coloring and flavoring ingredients (tomato/pepper powder or extract, etc.) seems promising for the future research.

## Figures and Tables

**Table 1:** Consequences of sterilization and storage on sterilized dairy food quality.

Consequence of Sterilization and/or Storage	Type of Sterilized Food Product	References
Amino acid losses	Processed cheese	[12,20,55]
Available lysine decrease	Processed cheese	[19]
Ammonia increase	Processed cheese	[8,9,12,19]
Protein profile changes	Processed cheese	[12,20]
	UHT milk	[69]
Lipid oxidation reactions	Processed cheese	[8,9]
	UHT milk	[100]
	UHT milk	[101]
Fat globules changes	Processed cheese	[12,77,103]
	Cream	[78,79]
	Cream cheese	[73]
	UHT milk	[101]
Antioxidant capacity reduction	UHT milk	[104]
Isomerization of lactose to lactulose	UHT milk	[74,83,106]
Vitamin losses	UHT milk	[13,108,109,110]
Calcium solubility/absorption reduction	UHT milk	[112]
Color changes (darkening)	Processed cheese	[8,9,12,19,20,70,71,72]
	UHT milk	[74,88,89,90]
	Cream cheese	[73]
	Concentrated milk	[75]
Consistency changes (hardening, thickening)	Processed cheese	[8,9,71,77]
	Cream	[78,79]
	UHT milk	[74,89]
	Concentrated milk	[75]
Stability impairment	Cream	[80]
	Infant formula emulsion	[81]
	UHT milk	[74,90,91]
	Concentrated milk	[82]
Sensory quality deterioration	Processed cheese	[8,9,19,20,84]
	UHT milk	[83,90]

## Data Availability

No new data were created or analyzed in this study. Data sharing is not applicable to this article.

## Abbreviations

The following abbreviations are used in this manuscript:

AA	Amino acid
FA	Fatty acid
LOR	Lipid oxidation reaction
MO	Microorganism
MRC	Maillard reaction complex
PC	Processed cheese
SDS-PAGE	Sodium dodecyl sulfate polyacrylamide gel electrophoresis
SPC	Sterilized processed cheese
TBARS	Thiobarbituric acid reactive substances
UHT	Ultra-high temperature

## References

[1] Tamime A.Y., Tamime A.Y. (2011). Processed cheese and analogues: An overview. Processed Cheese and Analogues.

[2] Guinee T.P., Mc. Sweeney P.L.H., Fox P.F., Cotter P.D., Everett D.W. (2017). Pasteurized processed and imitation cheese products. Cheese—Chemistry, Physics and Microbiology.

[3] Kapoor R., Metzger L.E. (2008). Processed cheese: Scientific and technological aspects—A review. Compr. Rev. Food Sci. Food Saf..

[4] Buňka F., Salek R.N., Kůrová V., Buňková L., Lorencová E. (2024). The impact of phosphate- and citrate-based emulsifying salts on processed cheese techno-functional properties: A review. Int. Dairy J..

[5] Deshwal G.K., Gomez-Mascaraque L.G., Fenelon M., Huppertz T. (2023). A review on the effect of calcium sequestering salts on casein micelles: From model milk protein systems to processed cheese. Molecules.

[6] Regulation (EC) No 1333/2008 of the European Parliament and of the Council of 16 December 2008 on Food Additives. https://eur-lex.europa.eu/legal-content/CS/TXT/?uri=celex%3A32008R1333.

[7] Schär W., Bosset J.O. (2002). Chemical and physico-chemical changes in processed cheese and ready-made fondue during storage—A review. LWT.

[8] Buňka F., Sedlačík M., Foltin P., Lazárková Z., Pětová M., Buňková L., Purevdorj K., Talár J., Kůrová V., Novotný M. (2023). Evaluation of processed cheese viscoelastic properties during sterilization observed in situ. J. Dairy Sci..

[9] Jedounková A., Lazárková Z., Hampelová L., Kůrová V., Pospiech M., Buňková L., Foltin P., Salek R.N., Malíšek J., Michálek J. (2022). Critical view on sterilisation effect on processed cheese properties designed for feeding support in crisis and emergency situations. LWT.

[10] Yang T.C.S., Barret A.H., Cardello A.V. (2012). Thermal Processing of Rations. Military Food Engineering and Ration Technology.

[11] Wang W.Z., Chen H.M., Ke D.M., Chen W.X., Zhong Q.P., Chen W.J., Yun A.Y.H. (2020). Effect of sterilization and storage on volatile compounds, sensory properties and physicochemical properties of coconut milk. Microchem. J..

[12] Bubelová Z., Tremlová B., Buňková L., Pospiech M., Vítová E., Buňka F. (2015). The effect of long-term storage on the quality of sterilized processed cheese. J. Food Sci. Technol..

[13] Gliguem H., Birlouez-Aragon I. (2005). Effects of sterilization, packaging, and storage on vitamin C degradation, protein denaturation, and glycation in fortified milks. J. Dairy Sci..

[14] Tulach P., Foltin P., Dujak D. (2019). Research methods in humanitarian logistics—Current approaches and future trends. Proceedings of the 19th International Scientific Conference Business Logistics in Modern Management.

[15] Pavlík V., Šafka V. (2022). Nové balené potravinové dávky v Armádě České republiky. Mil. Med. Sci. Lett..

[16] (2019). Requirements of Individual Operational Rations for Military Use Version 5.

[17] (2019). AMedP-1.11.

[18] Foltin P., Brunclík M., Ondryhal V., Vogal L. (2018). Usability of performance indicators of logistics infrastructure availability in supply chain designing. Bus. Log. Modern Manag..

[19] Lazárková Z., Buňka F., Buňková L., Valášek P., Kráčmar S., Hrabě J. (2010). Application of different sterilising modes and the effect on processed cheese quality. Czech J. Food Sci..

[20] Lazárková Z., Buňka F., Buňková L., Holáň F., Kráčmar S., Hrabě J. (2011). The effect of different heat sterilization regimes on the quality of canned processed cheese. J. Food Process Eng..

[21] Mulsow B.B., Jaros D., Rohm H., Tamime A.Y. (2007). Processed cheese and cheese analogues. Structure of Dairy Products.

[22] de Oliveira M.N., Ustunol Z., Tamime A.Y., Tamime A.Y. (2011). Manufacturing practices of processed cheese. Processed Cheese and Analogues.

[23] Černíková M., Buňka F., Salek R.N., El-Bakry M., Mehta B.M. (2022). Technological aspects of processed cheese: Properties and structure. Processed Cheese Science and Technology.

[24] Ozturk M., Kilic-Akyilmaz M., El-Bakry M., Mehta B.M. (2022). Manufacture of processed cheese: Equipments used. Processed Cheese Science And Technology.

[25] Buňková L., Buňka F. (2017). Microflora of processed cheese and the factors affecting it. Crit. Rev. Food Sci. Nutr..

[26] Onyeaka H.N., Nwabor O.F. (2022). Food Preservation and Safety of Natural Products.

[27] Shaker A.S., Ali M.A., Fathy H.M., Marrez D.A. (2022). Food preservation: Comprehensive overview of techniques, applications and hazards. Egypt. J. Chem..

[28] Erkmen O., Bozoglu T.F. (2016). Food Microbiology: Principles into Practice.

[29] Stopforth J.D., Juneja V.K., Dwivedi H.P., Sofos J.N. (2017). Preservation methods for meat and poultry. Microbial Control and Food Preservation—Theory and Practise.

[30] Lücke F.-K., Zeuthen P., Bøgh-Sørensen L. (2003). The control of pH. Food Preservation Techniques.

[31] Holdsworth S.D., Richardson P. (2004). Optimising the safety and quality of thermally processed packaged foods. Improving the Thermal Processing of Foods.

[32] Bown G., Richardson P. (2004). Modelling and optimising retort temperature control. Improving the Thermal Processing of Foods.

[33] Mukhopadhayay S., Ukuku D.O., Juneja V.K., Nayak B., Olanya M., Juneja V.K., Dwivedi H.P., Sofos J.N. (2017). Principles of food preservation. Microbial Control and Food Preservation—Theory and Practise.

[34] Bown G., Zeuthen P., Bøgh-Sørensen L. (2003). Developments in conventional heat treatment. Food Preservation Techniques.

[35] Oliveira J.C., Richardson P. (2004). Optimising the efficiency and productivity of thermal processing. Improving the Thermal Processing of Foods.

[36] Singh R.P., Heldman D.R. (2014). Introduction to Food Engineering.

[37] Fellows P.J. (2022). Food Processing Technology: Principles and Practice.

[38] Ramesh M.N., Rahman M.S. (2007). Canning and sterilization of foods. Handbook of Food Preservation.

[39] Deak T., Farkas J. (2013). Microbiology of Thermally Preserved Foods—Canning and Novel Physical Methods.

[40] Stojanović B., Janković S., Đorđević V., Marjanović S., Vasilev D., Stojanović Z., Balaban M., Antić V. (2021). Determination of toxic elements in meat products from Serbia packaged in tinplate cans. Environ. Sci. Pollut. Res..

[41] Stojanović B., Vasilev D., Stojanović Z., Parunović N., Janković S., Stanojević S., Balaban M., Antić V. (2021). Determination of sensory properties and levels of trace elements during storage of canned meat products. J. Food Process. Preserv..

[42] Jimenez P.S., Bangar S.P., Suffern M., Whiteside W.S. (2024). Understanding retort processing: A review. Food Sci. Nutr..

[43] Simpson R., Richardson P. (2004). Optimising the efficiency of batch processing with retort systems in thermal processing. Improving the Thermal Processing of Foods.

[44] den Besten H.M.W., Wells-Bennik M.H.J., Zwietering M.H. (2018). Natural diversity in heat resistance of bacteria and bacterial spores: Impact on food safety and quality. Annu. Rev. Food Sci. Technol..

[45] Berk Z. (2018). Food Process Engineering and Technology.

[46] Peleg M., Zeuthen P., Bøgh-Sørensen L. (2003). Modelling applied to process: The case of thermal preservation. Food Preservation Techniques.

[47] Heldman D.R. (2011). Food Preservation Process Design.

[48] Sarker D.K. (2020). Packaging Technology and Engineering: Pharmaceutical, Medical and Food Applications.

[49] Deák T., Motarjemi Y., Moy G., Todd E. (2014). Food Technologies: Sterilization. Encyclopedia of Food Safety.

[50] Tucker G.S., Richardson P. (2004). Validation of heat process: An overview. Improving the Thermal Processing of Foods.

[51] Shaw G.H., Richardson P. (2004). The use of data loggers to validate thermal processes. Improving the Thermal Processing of Foods.

[52] Rufe P.D. (2013). Fundamentals of Manufacturing.

[53] Jongen W. (2002). Fruit and Vegetable Processing—Improving Quality.

[54] Kristensen D., Skibsted L.H. (1999). Comparison of three methods based on electron spin resonance spectrometry for evaluation of oxidative stability of processed cheese. J. Agric. Food Chem..

[55] Buňka F., Hrabě J., Kráčmar S. (2004). The effect of sterilisation on amino acid contents in processed cheese. Int. Dairy J..

[56] Murata M. (2021). Browning and pigmentation in food through the Maillard reaction. Glycoconj. J..

[57] Xiang J., Liu F., Wang B., Chen L., Liu W., Tan S. (2021). A literature review on maillard reaction based on milk proteins and carbohydrates in food and pharmaceutical products: Advantages, disadvantages, and avoidance strategies. Foods.

[58] Lund M.N., Ray C.A. (2017). Control of Maillard reactions in foods: Strategies and chemical mechanisms. J. Agric. Food Chem..

[59] Poojary M., Lund N. (2022). Chemical stability of proteins in foods: Oxidation and the Maillard reaction. Annu. Rev. Food Sci. Technol..

[60] Aalaei K., Rayner M., Sjöholm I. (2018). Chemical methods and techniques to monitor early Maillard reaction in milk products: A review. Crit. Rev. Food Sci. Nutr..

[61] Thorpe S.R., Baynes J.W. (2003). Maillard reaction products in tissue proteins: New products and new perspectives. Amino Acids.

[62] Zhou Z., Langrish T. (2021). A review of Maillard reactions in spray dryers. J. Food Eng..

[63] Pizzoferrato L., Manzi P., Vivanti V., Nicoletti I., Corradini C., Cogliandro E. (1998). Maillard reaction in milk-based foods: Nutritional consequences. J. Food Prot..

[64] Starowicz M., Zieliński H. (2019). How Maillard reaction influences sensorial properties (color, flavor and texture) of food products?. Food Rev. Int..

[65] Iriondo-DeHond A., Elizondo A.S., Iriondo-DeHond M., Ríos M.B., Mufari R., Mendiola J.A., Ibanez E., Del Castillo M.D. (2020). Assessment of healthy and harmful Maillard reaction products in a novel coffee cascara beverage: Melanoidins and acrylamide. Foods.

[66] Li Y., Wu Y.R., Quan W., Jia X.D., He Z.Y., Wang Z.J., Adhikari B., Chen J., Zeng M.M. (2021). Quantitation of furosine, furfurals, and advanced glycation end products in milk treated with pasteurization and sterilization methods applicable in China. Food Res. Int..

[67] Ferrer E., Alegría A., Farré R., Abellán P., Romero F. (2000). Effects of thermal processing and storage on available lysine and furfural compounds contents of infant formulas. J. Agric. Food Chem..

[68] Torbatinejad N.M., Rutherfurd S.M., Moughan P.J. (2005). Total and reactive lysine contents in selected cereal-based food products. J. Agric. Food Chem..

[69] Gaucheron F., Mollé D., Briard V., Léonil J. (1999). Identification of low molar mass petides, released during sterilisation of milk. Int. Dairy J..

[70] Daly D.F.M., McSweeney P.L.H., Sheehan J.J. (2012). Pink discolouration defect in commercial cheese: A review. Dairy Sci. Technol..

[71] Buňka F., Štětina J., Hrabě J. (2008). The effect of storage temperature and time on the consistency and colour of sterilized processed cheese. Eur. Food Res. Technol..

[72] Fann J., Zhang J., Li H., Jiang S., Gao Y., Zhang L. (2014). Study of methods of inhibiting browning of stored sterilized processed cheese. J. Chin. Ins. Food Sci. Technol..

[73] Song B., Zhu P., Zhang Y., Ju N., Si X., Pang X., Lv J., Zhang S. (2023). Preparation and quality assessment of processed cream cheese by high hydrostatic pressure combined thermal processing and spore-induced germination. J. Food Eng..

[74] Ranvir S., Sharma R., Gandhi K., Nikam P., Mann B. (2021). Physico-chemical changes during processing and storage of UHT milk. Indian J. Dairy Sci..

[75] Balde A., Aider M. (2019). Impact of sterilization and storage on the properties of concentrated skim milk by cryoconcentration in comparison with vacuum evaporation and reverse osmosis concentration. J. Food Process Eng..

[76] Friedman M. (1996). Food browning and its prevention: An overview. J. Agric. Food Chem..

[77] Li H., Wu Y., Hou D., Zhao S., Li D., Wang X., Li H., Yu J. (2023). Effects of pre-emulsification with whey protein and high temperature sterilisation on texture, functional characteristics and Maillard reaction products of room temperature stored processed cheese. Int. Dairy J..

[78] Long Z., Zhao M., Sun-Waterhouse D., Lin Q., Zhao Q. (2016). Effects of sterilization conditions and milk protein composition on the rheological and whipping properties of whipping cream. Food Hydrocoll..

[79] Wang S., Li Y., Yan G., Yuan D., Ji B., Zhou F., Li Y., Zhang L. (2023). Thickening mechanism of recombined dairy cream stored at 4 °C: Changes in the composition and structure of milk protein under different sterilization intensities. Int. J. Biol. Macromol..

[80] Dhungana P., Truong T., Bansal N., Bhandari B. (2019). Apparent thermal and UHT stability of native, homogenized and recombined creams with different average fat globule sizes. Food Res. Int..

[81] McSweeney S.L., Mulvihill D.M., O’Callaghan D.M. (2004). The influence of pH on the heat-induced aggregation of model milk protein ingredient systems and model infant formula emulsions stabilized by milk protein ingredients. Food Hydrocoll..

[82] Dumpler J., Huppertz T., Kulozik U. (2020). Invited review: Heat stability of milk and concentrated milk: Past, present, and future research objectives. J. Dairy Sci..

[83] Valero E., Villamiel M., Miralles B., Sanz J., Martínez-Castro I. (2001). Changes in flavour and volatile components during storage of whole and skimmed UHT milk. Food Chem..

[84] Bertrand E., Machado-Maturana E., Chevarin C., Portanguen S., Mercier F., Tournayre P., Abouelkaram S., Guillard A.-S., Kondojoyan A., Berdafué J.-L. (2011). Heat-induced volatiles and odour-active compounds in a model cheese. Int. Dairy J..

[85] Rannou C., Laroque D., Renault E., Prost C., Sérot T. (2016). Mitigation strategies of acrylamide, furans, heterocyclicamines and browning during the Maillard reactions in foods. Food Res. Int..

[86] Adamiec J., Cejepk K., Rössner J., Velíšek J. (2001). Novel Strecker degradation products of tyrosine and dihydroxyphenylalanine. Czech J. Food Sci..

[87] Kristensen D., Hansen E., Arndal A., Trinderup R.A., Skibsted L.H. (2001). Influence of light and temperature on the colour and oxidative stability of processed cheese. Int. Dairy J..

[88] Popov-Raljić J.V., Lakić N.S., Laličić-Petronijević J.G., Barać M.B., Sikimić V.M. (2008). Color Changes of UHT Milk During Storage. Sensors.

[89] Zeren R.B., Buzrul S., Bilge G. (2024). Monitoring the changes in UHT whole milk during storage under dynamic and constant temperature profiles. J. Food Sci. Technol..

[90] Karlsson M.A., Langton M., Innings F., Malmgren B., Höjer A., Wikström M., Lundh Å. (2019). Changes in stability and shelf-life of ultrahigh temperature treated milk during long term storage at different temperatures. Heliyon.

[91] Malmgren B., Ardö Y., Langton M., Altskär A., Bremer M.G.E.G., Dejmek P., Paulsson M. (2017). Changes in proteins, physical stability and structure in directly heated UHT milk during storage at different temperatures. Int. Dairy J..

[92] Al-Saadi J.M.S., Deeth H.C. (2008). Cross-linking of proteins and other changes in UHT milk during storage at different temperatures. Austral. J. Dairy Technol..

[93] Turner N.A., Vulfson E.N. (2000). At what temperature can enzymes maintain their catalytic activity?. Enzyme Microb. Technol..

[94] Haki G.D., Rakshit S.K. (2003). Developments in industrially important thermostable enzymes: A review. Biores. Technol..

[95] Synowiecki J., Grzybowska B., Zdziebło A. (2006). Sources, properties and suitability of new thermostable enzymes in food processing. Crit. Rev. Food Sci. Nutr..

[96] Domínguez R., Pateiro M., Gagaoua M., Barba F.J., Zhang W., Lorenzo J.M. (2019). A comprehensive review on lipid oxidation in meat and meat products. Antioxidants.

[97] Geng L., Liu K., Zhang H. (2023). Lipid oxidation in foods and its implications on proteins. Front. Nutr..

[98] Zeng J., Song Y., Fan X., Luo J., Song J., Xu J., Xue C. (2023). Effect of lipid oxidation on quality attributes and control technologies in dried aquatic animal products: A critical review. Crit. Rev. Food Sci. Nutr..

[99] Wagner K.-H., Auer E., Elmadfa I. (2000). Content of trans fatty acids in margarines, plant oils, fried products and chocolate spreads in Austria. Eur. Food Res. Technol..

[100] Ajmal M., Nadeem M., Imran M., Junaid M. (2018). Lipid compositional changes and oxidation status of ultra-high temperature treated milk. Lipids Health Dis..

[101] Li Y.H., Wang W.J., Zhang F., Shao Z.P., Guo L. (2019). Formation of the oxidized flavor compounds at different heat treatment and changes in the oxidation stability of milk. Food Sci. Nutr..

[102] Luna P., Angel De La Fuente M.A., Juárez M. (2005). Conjugated linoleic acid in processed cheeses during the manufacturing stages. J. Agric. Food Chem..

[103] Tremlová B., Štarha P., Buňka F., Gistingrová Z., Hrabě J. (2006). The effect of sterilization on size and shape of fat globules in model processed cheese samples. Acta Vet. Brno.

[104] Dias F.F.G., Augusto-Obara T.R., Hennebelle M., Chantieng S., Ozturk G., Taha A.Y., Ferreira de Souza Vieira T.M., Nobrega de Moura Bell J.M.L. (2020). Effects of industrial heat treatments on bovine milk oxylipins and conventional markers of lipid oxidation. Prostaglandins Leukot. Essent. Fat. Acids.

[105] Ajandouz E.H., Tchiapke L.S., Dalle Ore F., Benajiba A., Puigserver A. (2001). Effects of pH on caramelization and Maillard reaction kinetics in fructose-lysine model systems. J. Food Sci..

[106] de Oliveira Neves L.N., De Oliveira M.A.L. (2020). Determination of lactose and lactulose isomers in UHT milk by CZE-UV. LWT.

[107] Ryley J., Kajda P. (1994). Vitamins in thermal processing. Food Chem..

[108] Pingali A.V., Trumbo P.R. (1992). Effect of sterilization of milk on vitamin B-6 composition and bioavailability. J. Agric. Food Chem..

[109] Sharma R., Lal D. (1998). Influence of various heat processing treatments on some B-vitamins in buffalo and cow’s milks. J. Food Sci. Technol..

[110] Sachdeva B., Kaushik R., Arora S., Khan A. (2021). Effect of processing conditions on the stability of native vitamin A and fortified retinol acetate in milk. Int. J. Vitam. Nutr. Res..

[111] Kaushik R., Sachdeva B., Arora S. (2014). Vitamin D_2_ stability in milk during processing, packaging and storage. LWT.

[112] Seiquer I., Delgado-Andrade C., Haro A., Navarro M.P. (2010). Assessing the effects of severe heat treatment of milk on calcium bioavailability: In vitro and in vivo studies. J. Dairy Sci..

